# Polycaprolactone–Itaconic Acid Resins for Additive Manufacturing of Environmentally Degradable 3D and 4D Materials by Thiol-ene Photopolymerization

**DOI:** 10.1021/acs.macromol.5c01310

**Published:** 2025-08-05

**Authors:** Bo Li, Gianluca Bartolini Torres, Baptiste Martin, Nicholas Taylor, Eugen Barbu, Annette Christie, Andreas Heise

**Affiliations:** † Department of Chemistry, 8863 RCSI University of Medicine and Health Sciences, Dublin D02 YN77, Ireland; ‡ Science Foundation Ireland (SFI) Centre for Research in Medical Devices (CURAM), RCSI, Dublin D02 YN77, Ireland; § AMBER, The SFI Advanced Materials and Bioengineering Research Centre, RCSI, Dublin D02 YN77, Ireland; ∥ Laboratoire de Chimie de Coordination, CNRS, 54919 Université de Toulouse (UPS, INP), 31077 Toulouse, France; ⊥ Jealott’s Hill International Research Centre, 101825 Syngenta, Bracknell RG42 6EY, U.K.

## Abstract

Digital light processing (DLP) has emerged as a powerful tool for advanced manufacturing, enabling the fabrication of intricate 3D polymer structures and, more recently, responsive 4D architectures that adapt to environmental stimuli. However, current DLP technologies rely heavily on acrylate-based photocurable resins, which pose significant sustainability challenges from resin synthesis to end-of-life disposal. To address these issues, we present a novel solvent-free approach to functionalizing polycaprolactone (PCL) using biomass-derived itaconic acid (IA). The unsaturated moiety of IA enables efficient photopolymerization via thiol-ene chemistry in both dioxane and the sustainable solvent γ-valerolactone, affording excellent printability. In the resulting cross-linked networks, IA end-groups serve not only as photocurable sites but also as functional handles that confer environmental responsiveness, as demonstrated by pH-triggered 4D transformations and dye uptake. To simulate end-of-life conditions, we demonstrated hydrolysis and microbial degradation of the cross-linked materials in a sewage-derived inoculum, supporting the potential for biomass regeneration in a circular materials framework. This strategy provides a sustainable route to producing functional, mechanically robust resins for 3D and 4D printing, offering a reduced environmental impact without compromising performance.

## Graphical abstract

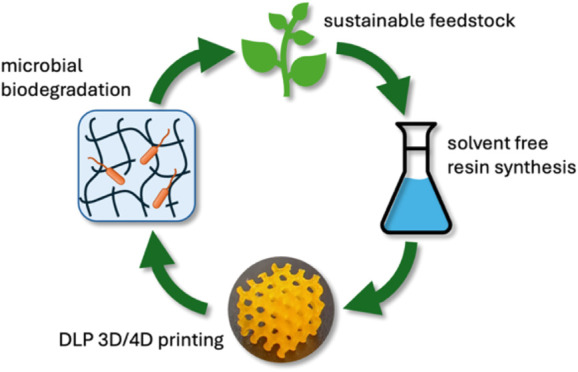

## Introduction

Digital light processing (DLP) is a rapidly developing additive manufacturing technique allowing high printing speed and resolution of 3D structures.^1–4^ Recently, the technology has further advanced from static 3D structures to 4D materials, which can undergo programmed transformations once exposed to specific triggers.^5,6^ Examples include DLP-printed polymers utilizing dynamic bonds to achieve materials with adaptable size and mechanical strength or sugar-responsive hydrogel microstructures from two-photon 3D printing, among many others.^7,8^ As DLP prints through vat polymerization, photoreactive materials such as (meth)­acrylates and (meth)­acrylated polymers are commonly used as base resins.^9–12^ Depending on the choice of resins, 3D and 4D structures with various properties can be printed for the desired applications.^13–16^ However, most reported DLP procedures encounter sustainability challenges at every stage of the process, including material selection, resin synthesis, and the end-of-life management of the printed product.^17–20^ Overcoming these challenges and developing sustainable processes, while preserving the material properties and resolution of 3*D*/4D structures, is essential to minimizing the future environmental impact of additive manufacturing.^21^ While achieving complete sustainability at every level may not always be feasible, the goal should be to minimize environmental impact as much as possible.

Some renewable feedstocks such as terpenes, fumaric acid, and microalgae-derived triglycerates have been investigated as potential candidates for vat 3D printing.^22–24^ However, many of these feedstocks require chemical modification to achieve photoreactivity, often involving harmful reagents.^25,26^ Similarly, highly cross-linked networks formed from acrylates (whether monomers or polymers) present end-of-life challenges due to their nondegradability. To address this, reversible chemistries such as cycloaddition and thiol-ene reactions have been explored as viable alternatives in DLP printing.^27–29^ The thiol-ene reaction differs from free-radical polymerization (FRP) in that it proceeds via a step-growth mechanism, leading to more uniform and defect-free networks. The mechanical properties of thiol-ene networks therefore offer improved control and reduced shrinkage, compared to the inhomogeneous networks of FRP.^30–32^ Resins cross-linked with these chemistries can be depolymerized under light or temperature and, in some cases, be reprinted. For example, a recent study has reported a fully recyclable resin derived from renewably sourced lipoic acid.^33^ However, many of the reported materials are synthesized via nongreen methods, potentially undermining their overall sustainability, particularly at an industrial scale, despite a recent 4D printing roadmap highlighting the need for alignment with sustainability goals.^34^

We present a protocol for printing 3D and 4D materials that achieves a high level of sustainability across all processing stages from resin synthesis to end-of-life management (Figure 1). The resulting materials possess properties suitable for a wide range of applications, along with a programmable pH response as 4D materials. The resin is derived from itaconic acid and polycaprolactone (PCL), a biodegradable polymer. While the commercial monomer caprolactone (CL) is petrochemically derived, recent reports demonstrated that it can be sourced from biomass, classifying it as a future potential renewable feedstock.^21,35^ PCL is widely used in 3D printing for applications such as tissue engineering.^36–39^ For this, PCL is typically functionalized with reactive double bonds using hazardous chemicals like isocyanates, acid chlorides, and excess solvents for synthesis and purification.^30,40^ To overcome this issue, we propose a PCL resin using biomass-derived itaconic acid (IA) as a reactive end-capper. While IA has previously been used in 3D printing,^41–44^ it is still under-utilized in vat 3D printing due to its low reactivity in radical polymerization,^45,46^ caused by the steric hindrance of its double bond. In one recent example, functionalizing PCL with itaconate was reported.^47^ However, the complex synthesis with methyl itaconyl chloride required chlorination agents and excess solvents, offering very limited improvement in sustainability. Moreover, the free-radical DLP involved acrylic comonomers and extensive postcuring due to the low reactivity of the itaconate double bond. We devised a process in which photoreactive polymers are obtained in a solvent-free protocol, combining the ring-opening polymerization of CL with selective esterification using IA in a single processing step. We demonstrate the excellent printability of the PCL-itaconic acid (PCL–IA) resin in DLP through thiol-ene chemistry using dioxane and a green solvent, γ-valerolactone (GVL), into 3D structures. Moreover, pH-responsive 4D hydrogel from PCL–IA and PEG thiol cross-linker was printed for the first time without the need for a comonomer. To simulate end-of-life scenarios, the microbial biodegradation of the cross-linked materials was evaluated in sewage water-derived inoculum, which served as a model for regenerating biomass feed from the printed materials in a circular process. Our approach offers a straightforward and scalable pathway for producing functional mechanically robust resins for 3D and 4D printing, which marks a significant advancement in addressing the sustainability challenges of additive manufacturing.

**Figure 1:**
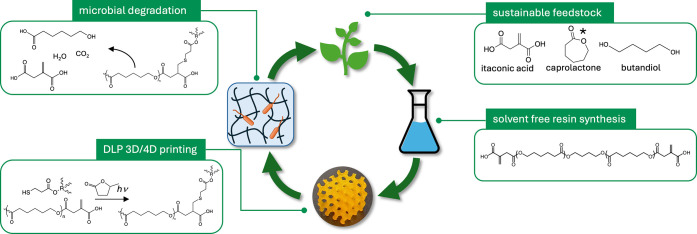
Approach to sustainable 3D and 4D structures. *Caprolactone synthesis from sustainable feedstock has been demonstrated but is not commercially implemented to date.

## Experimental Procedure

### Materials

ε-Caprolactone, γ-valerolactone (GVL), toluene, and benzene were purchased from TOKYO CHEMICAL INDUSTRY. Tin­(II) octoate, 1,4-butanediol (BDO), itaconic acid, pentaerythritol tetrakis­(3-mercaptopropionate), phenylbis­(2,4,6-trimethylbenzoyl)­phosphine oxide (BAPO), sodium hydroxide, dimethylformamide (DMF), 1,4-dioxane, methanol, 1,8-diazabicyclo(5.4.0)­undec-7-ene (DBU), sulfuric acid, 3-mercaptopropionic acid, dichloromethane, NaHCO_3_, and M9 minimal salts were purchased from Sigma-Aldrich. MgSO_4_ was purchased from Fluorochem. Four-arm PEG2K-OH was purchased from JENKEM TECHNOLOGY USA. 4-arm PEG-SH was synthesized following a literature procedure.^48^ Allylthiourea (ATU) solution (5 g/L in water) was purchased from WTW/Xylem Analytics and used as received.

### Methods

^1^H and ^1^H diffusion-ordered spectroscopy (DOSY) spectra were recorded on a Bruker Advance 400 MHz instrument at room temperature. All chemical shifts (δ) were reported in parts per million (ppm) and analyzed relative to the residual nuclei of the deuterated solvent, while diffusion coefficients were reported in cm^2^ s^–1^. Size exclusion chromatography (SEC) was performed on a CHCl_3_ Agilent Technologies LC 1200 Series equipped with an Agilent 1260 ISO pump, Agilent refractive index detector, and SDV 5 μm 8 × 50 mm precolumn and 2 SDV 5 μm 8 × 300 mm columns in series were used to determine molecular weight distributions and polydispersity indexes. The chromatograms were recorded with a flow rate of 1.0 mL min^–1^ at 40 °C. The system was calibrated with PSS Polymer Standards Service GmbH linear poly­(methyl methacrylate). Samples were dissolved in CHCl_3_ at a concentration of 4 mg mL^–1^ and filtered through a 0.2 μm Millipore filter prior to injection. A Thorlabs 405 nm UV LED light (M405L3-C1) was used to photocure the resins. An Anton Paar MCR 301 equipped with a parallel plate of 25 mm diameter was used to conduct rheology experiments. A gap length of 0.05 mm was used for all of the rheometer experiments at room temperature. Photorheology experiments were carried out using a rheometer equipped with 405 nm UV light and a sample glass plate to allow the passage of light. A Peltier hood was used to protect the sample from ambient light. Data points were collected every 10 s through a time sweep experiment with constant oscillation at 10 rad s^–1^ with a strain of 0.1%. UV light (9 mW cm^–2^ was turned on after 60 s). Attenuated total reflectance Fourier Transform Infrared (ATR-FTIR) was carried out on a PerkinElmer Spectrum 100 in the spectral range of 500–4000 cm^–1^. Dynamic mechanical analysis (DMA) was carried out on a DMA 850 from TA Instruments in tension mode. Oscillation measurements were carried out with a frequency of 1 Hz and a strain of 0.08%, while temperature varied at 3 °C/min from −100 to 100 °C. For sample preparation, each resin was cast into a Petri dish and closed. Then, it was irradiated with UV light at 405 nm (2 mW cm^–2^) for 1 h at room temperature. Afterward, the solvent was removed by evaporation, and the resulting film was cut into rectangles of *L* 20 mm × *W* 10 mm, having a thickness of ca. 0.4 mm. A Testometric M100–1CT machine equipped with a 50 N cell load (LC50) was used to perform tensile tests at room temperature. A gauge length of 8 mm, test speed of 10 mm mm^–1^, and pretension of 0.1 N were set as machine parameters. Samples were prepared by pouring each resin into rectangular molds (*H* 0.8 mm × *L* 20 mm × *W* 10 mm) and irradiated for 1 h at room temperature with UV light at 405 nm (2 mW cm^–2^). Afterward, the solvent was removed by evaporation to obtain sheets of size *H* 0.4–0.5 mm × *W* 7–8 mm × *L* 14–16 mm. A TA Instruments DSC Q200 and TA Instruments RSC FC-100 immersion cooler were used to perform the differential scanning calorimetry (DSC) experiments. A heating ramp of 10 °C per minute was used from −50 to 100 °C for two cycles. A mass of 5–10 mg of dry sample was used for each measurement. Each sample was measured in an aluminum Tzero pan under nitrogen flow using an empty pan as a reference. A custom MONO3-2K40 from Monoprinter (US) equipped with UV LED 405 nm was used for digital light processing (DLP) 3D printing. The projector resolution was 1902 × 1080 pixels with an in-plane resolution of 15 μm. A layer thickness of 50 μm and light intensity of 22 mW cm^–2^ (measured on the tank surface) were used for each printing at room temperature with an irradiation time of 22 s. The CAD design for the gyroid structure was generated using the software MSLattice. The CAD design for the gear was generated using the program Tinkercad (tinkercad.com) by Autodesk.

### Synthesis of PCL–IA

Polycaprolactone was synthesized via ring-opening polymerization of ε-caprolactone (5 g, 44 mmol, 18 equiv) using 1,4-butanediol (BDO, 0.219 g, 2.4 mmol, 1 equiv) as an initiator and Tin­(II) octoate (0.029 g, 0.072 mmol, 0.03 equiv) as a catalyst. The reaction mixture was stirred under a nitrogen atmosphere at 110 °C for 16 h. Subsequently, PCL was chain-end functionalized by adding itaconic acid (0.624 g, 4.8 mmol, 2 equiv) to the reaction mixture, followed by stirring under nitrogen at 140 °C for 18 h. After ca. 5 h, the reaction mixture became homogeneous and clear, indicating the reaction of the itaconic acid with the PCL chain ends. The polymer was used without further purification.

### Resin Formulation

General resin formulation consists of 23.5–27.5 wt % PCL–IA, 1.5–3.0 wt % pentaerythritol tetrakis­(3-mercaptopropionate) according to the thiol-ene molar ratio, 1.5 wt % BAPO, and 70 wt % 1,4-dioxane or γ-valerolactone. Sudan I (0.05 wt %) was added to the same formulation when the resin was intended for 3D printing. The resin formulation used for the formation of 4D hydrogel consists of 19.3 wt % of PCL–IA, 9.7 wt % of 4-arm-PEG-SH (2000), 1.5 wt % of BAPO, and 70 wt % of 1,4-dioxane. Sudan I (0.03 wt %) was added to the same formulation when resin was intended for printing.

### Methylene Blue Absorption Test

The test was performed on cross-linked discs of *H* 2 mm × Ø 7 mm size with a weight of ca. 100 mg (30 wt % polymer content in dioxane). Samples were prepared by pouring the resins PEG-SH:IA(1:1) into a disc-shaped mold and irradiating with 405 nm UV light (2 mW·cm^–2^) for 1 h at room temperature. The freshly formed organogels were directly immersed in buffer solution at different pH (4, 7, 10), each containing a concentration of methylene blue at 7.5 μg·mL^–1^. For each pH, the test was conducted at room temperature and with a swelling time of 24 h in triplicate. After that, the amount of unabsorbed methylene blue in the buffer solution was calculated via the UV–vis calibration curve (Figure S13). The difference with the initial concentration was calculated as the amount absorbed.

### Accelerated Degradation

A solution of 0.2 M NaOH was used to perform the degradation test at room temperature. Samples were prepared by pouring the resins into a disc-shaped mold and irradiating them with 405 nm UV light (2 mW cm^–2^) for 1 h at room temperature. The size of the disc was H 1.3 × Ø 5 mm after the removal of solvents and used without further treatment. Time points were taken every 2 h for the first 6 h and then every 3 h for the next 15 h, for a total period of 21 h. At each time point, samples were removed, rinsed with deionized water, dried, and then weighed. The degradation was calculated as the percentage of mass loss relative to the initial dry mass. The results were calculated as the average of three replicates.

### Biodegradation

Biodegradability of the cross-linked SH:IA (1:1) was determined using a manometric respirometry method based on the Organisation of Economic Cooperation and Development (OECD) 301F guidelines.^49^ The biochemical oxygen demand (BOD) for the test compound was measured over a period of 28 days using an OxiTop manometric measurement system (WTW, Germany), expressed as the drop in the internal pressure, Δ*p*, inside the measuring bottles from their initial equilibrated state. The percentage biodegradability, %*D*, was calculated as

1 $$\begin{array}{l} \%D=\left(\frac{\Delta p-\Delta p_{\mathrm{blank}}}{{\Delta p}_{\max}}\right)\times 100 \end{array}$$

Here, Δ*p*_blank_ is the averaged pressure drop of the inoculum blank bottles (corresponding to the background oxygen consumption of the bacterial inoculum without any test substance present), and Δ*p*_max_ is the pressure drop corresponding to 100% mineralization of the test substance and is calculated using

2 $$\begin{array}{l} {\Delta p}_{\max}=\frac{n_{O_{2}}\mathrm{RT}}{V_{h}}=\frac{m_{O_{2}}\mathrm{RT}}{M_{O_{2}}V_{h}}=\frac{{ThOD\times m}_{s}\mathrm{RT}}{M_{O_{2}}V_{h}} \end{array}$$

where *R* = gas constant (8.314 J K^–1^ mol^–1^), *T* = temperature (K), *m*_s_ = sample mass (g), *M*_O_2__ = molecular weight of oxygen (g mol^–1^), and *V*_h_ = headspace volume (m^3^).

ThOD is the theoretical oxygen demand of the test material (mg O_2_ per mg substance) required to fully oxidize/mineralize the sample and is calculated from the numbers of each atomic element in the empirical molecular formula using

3 $$\begin{array}{l} \mathrm{ThOD}=\left[\frac{16\times \left(2C+\frac{1}{2}\left(H-\mathrm{Cl}-3N\right)+3S+\frac{1}{2}\mathrm{Na}-O\right)}{M_{r}}\right] \end{array}$$

where *M*_r_ is the relative molecular mass of the repeating unit in the test substance.

The bacterial inoculum used for the biodegradation test was secondary effluent obtained from a local domestic wastewater treatment works (Bracknell Sewage Treatment Works, Thames Water, U.K.) and was used fresh on the day of receipt. The inoculum was filtered through a 10 μm syringe filter to remove coarse suspended solids (final solids content: ≤ 30 mg/L) and kept aerated by magnetic stirring at 20 ± 1 °C for 1–2 h prior to use. Duplicate samples were run for the cross-linked polymer samples, the positive control (poly­(3-hydroxybutyrate), PHB), and the inoculum blanks. The polymer samples and PHB were milled or ground to a powdered form prior to the experiment, and 10–12 mg of each test material was charged into a clean measurement bottle alongside a 4 cm magnetic stirrer bar. To each bottle, 108 mL of freshly prepared, sterilized M9 minimal medium (composition of 3 g/L KH_2_PO_4_, 0.4 g/L NaCl, 6.78 g/L Na_2_HPO_4_, and 1 g/L NH_4_Cl diluted into ultrapure water) was added, followed by two drops (∼0.1 mL) of a nitrification inhibitor (ATU solution – 5 g/L in water) and 12 mL of the filtered secondary effluent sample. After the addition of the inoculum, the bottles were promptly capped and sealed with a head assembly incorporating the OxiTop pressure measuring units and a small rubber thimble containing NaOH pellets to absorb the CO_2_ generated from mineralization of the test substance. The bottles were placed on a magnetic stirring platform inside a dark temperature-controlled incubator cabinet set to *T* = 25 (±0.5) °C, and the pressure drop inside the bottles was continuously measured over a period of 28 days. The measured pH of the samples was 7.4 ± 0.1 over the whole duration of the biodegradation test.

## Results and Discussion

### Solvent-free Synthesis of PCL–Itaconic Acid (PCL–IA)

To eliminate the use of solvents, the synthesis of PCL–IA was performed in bulk through a two-step, one-pot reaction (Figure 2A). Tin­(II)­octoate was chosen as the catalyst due to its effectiveness in both the ring-opening polymerization of CL and esterification reactions.^50,51^ 1,4-Butanediol (BDO) was selected as the initiator, given its widespread industrial biosynthesis production,^52^ aiming to produce a PCL diol with a target molar mass of 2100 g·mol^–1^ (monomer-to-initiator ratio of 18:1). Monomer conversion exceeded 99% after heating for 16 h at 110 °C under nitrogen, as indicated by the disappearance of the O=COC*H* (δ = 4.25 ppm) signal in the ^1^H NMR spectra (Figure S1). Subsequently, IA was added to the reaction mixture for chain-end functionalization of the PCL via esterification at 140 °C. It was hypothesized that transesterification reactions involving the IA diacid would be suppressed due to the low reactivity of its conjugate carboxylic acid groups, leading predominantly to IA-end-capped PCL. ^1^H NMR spectra taken at multiple time points confirmed complete consumption of IA after 18 h (Figure S2). The resulting polymer, recovered as a solid white material, was used without further purification. DOSY NMR revealed identical diffusion coefficients for IA and PCL, confirming their incorporation into a single molecule (Figure S3). The incorporation of IA as PCL chain ends was further confirmed by ^1^H NMR (Figure 2C), where IA signals corresponded to free carboxylic acid, consistent with a reaction of only one IA acid group. SEC analysis showed a slight increase in molecular weight after addition of the IA in agreement with the end-capping reaction (Figure 2B). While minor chain extension cannot be excluded, the data suggest quantitative PCL end-capping by IA additions. Overall, the results demonstrate that the solvent-free, one-pot procedure is effective for synthesizing IA-end-capped PCL without the need for additional workup.

**Figure 2:**
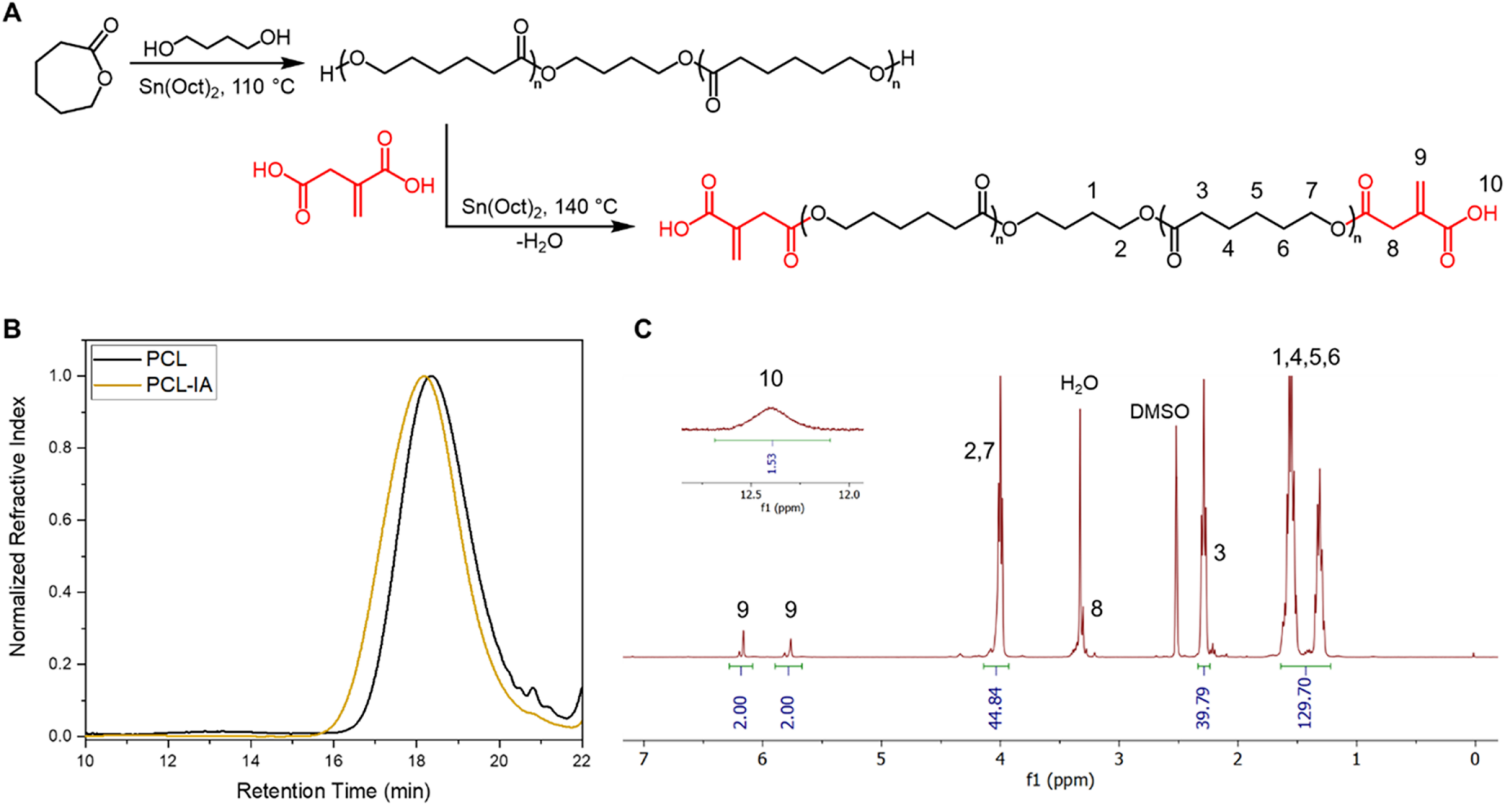
Scheme of a one-pot, two-step synthesis of PCL–IA (A). Size exclusion chromatography (SEC) results of PCL (*M*_n_ = 3300 g mol^–1^, *Đ* = 1.3) and PCL–IA (*M*_n_ = 3800 g mol^–1^, *Đ* = 1.6) in CHCl_3_ (dRI detection with PMMA standards) (B). ^1^H NMR spectrum of PCL–IA in DMSO-*d*_6_ (C).

### Development and Photoreactivity of PCL Resins

The unsaturated functional chain ends of PCL–IA were used to create cross-linked networks via thiol-ene chemistry, exploiting the high reactivity of the itaconic double bond toward thiol groups.^53^ The thiol-ene resin formulations consisted of PCL–IA, a tetra-thiol cross-linker (pentaerythritol tetrakis­(3-mercaptopropionate)), phenylbis­(2,4,6-trimethylbenzoyl)­phosphine oxide (BAPO) photoinitiator, and a nonreactive solvent. The choice of solvent was critical due to the differing polarities and moderate solubility of itaconic acid and PCL. Incorrect solvent selection could lead to chain-end self-assembly, which might interfere with the intended cross-linking. Following trials with various solvents, including renewable options in photorheology experiments (Figure S5), dioxane and γ-valerolactone (GVL), a sustainable solvent was chosen for further testing. The photoreactivity of the resins was then analyzed via photorheology using 405 nm UV light (9 mW cm^–2^) at room temperature, with a polymer concentration of approximately 26 wt % in dioxane. Resins with three different molar ratios of thiol (SH) to itaconic acid double bonds (IA) were tested to assess the thiol-ene reactivity (Table 1). While no clear gel point was observed for any of the tested resins, the divergence between the storage modulus (*G*′) and the loss modulus (*G*″) upon UV irradiation indicated the formation of cross-linked networks. The SH:IA (1:1) resin showed the fastest polymerization, with a higher *G*′_max_ of 9512 Pa compared to SH:IA (2:1) and SH:IA (1:2) formulations (Figure 3). As dioxane is considered hazardous, a SH:IA (1:1) sample was also tested in the green solvent GVL, where cross-linking was observed, but *G*′ was notably lower at 1022 Pa.^54^ When the tetra-thiol cross-linker was omitted, no significant network formation was observed in agreement with the low reactivity of the IA double bond in radical reactions.

**Figure 3:**
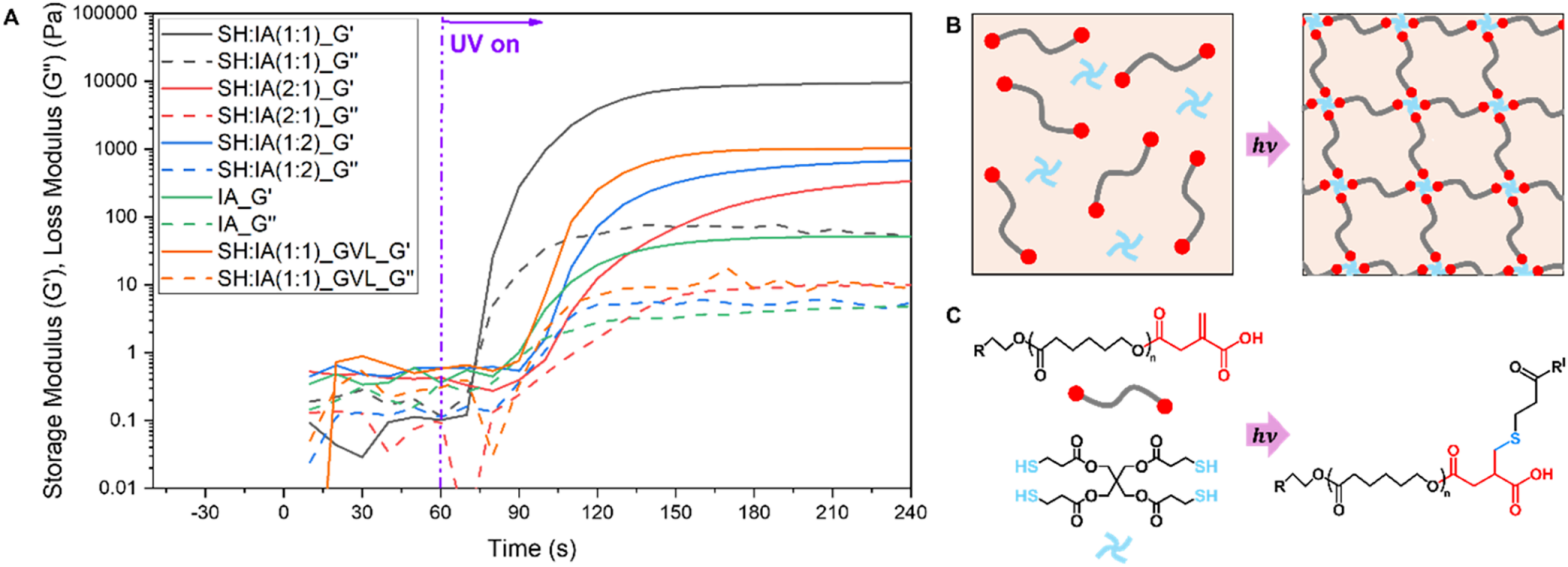
(A) Photorheology plots of the resin formulations listed in Table 1. UV light at 405 nm turned on at 60 s (9 mW cm^–2^). (B) Schematic representation of the network formation by thiol-ene. (C) Scheme of the thiol-ene reaction leading to the formation of a thiol-ether bond.

**Table 1:** Resin Formulations Based on PCL–IA with Varying Thiol-ene Ratios, Analyzed by Photorheology and Swelling Test^a^

resin	solvent	thiol [wt %]	polymer [wt %]	*G*′_max_ (240 s) [Pa]	gel fraction [%]	swelling ratio
SH:IA (1:1)	dioxane	3	26	9512	83.7 ± 1.3	4.6 ± 0.5
SH:IA (2:1)	dioxane	5.5	23.5	337	73.8 ± 1.5	6.8 ± 0.2
SH:IA (1:2)	dioxane	1.5	27.5	678	79.5 ± 0.7	6.2 ± 0.6
SH:IA (1:1)	GVL	3	26	1022	72.9 ± 1.2	8.0 ± 0.3
IA	dioxane		29	52	22.2 ± 10.1	74.3 ± 33.3

a All Dioxane Formulations Include 1.5 wt % BAPO Photoinitiator and GVL Formulation of 2 wt %.

Swelling tests conducted on samples cured in molds (Table 1) supported the photorheology results, with the SH:IA (1:1) sample exhibiting the lowest swelling and the highest gel content, indicating the most efficient cross-linking compared to the other ratios. For the SH:IA (2:1) network, the excess thiol groups likely led to the formation of competing disulfide bonds, reducing the overall cross-linking efficiency. In contrast, for the SH:IA (1:2) sample, the itaconic acid was not fully consumed by the thiol groups, resulting in lower cross-linking. When comparing the gel properties of SH:IA (1:1) in dioxane and GVL, an 11% lower gel content and higher swelling were observed in GVL, consistent with the photorheology data of a network with lower cross-linking density. This behavior may be attributed to the deprotonation of itaconic acid by the cyclic ester solvent and subsequent chain-end self-assembly.^55^

### Thermomechanical Properties

To assess the transition of the mechanical properties of the PCL–IA networks at different temperatures, dynamic mechanical thermal analysis (DMTA) was performed (Figure 4A). All samples were prepared using 1,4-dioxane as a solvent for consistent reactivity in the photorheology. The SH:IA (2:1) and SH:IA (1:2) networks exhibited similar glassy storage moduli under 3000 MPa, while the SH:IA (1:1) network showed a higher value of 3300 MPa. All networks demonstrated a distinct decrease in storage modulus, with glass transition temperatures (*T*_*g*_) ranging from −29 to −10 °C. The gradual decrease in storage modulus in the glass transition region is attributed to the semicrystalline nature of PCL in the thiol-ene networks. A second decrease in storage modulus was observed, corresponding to the melting of the networks, with melting temperatures ranging from 40 to 50 °C. The semicrystalline character of the networks was further confirmed by differential scanning calorimetry (DSC) (Figure S7). The mechanical properties of the networks were measured by static tests with a universal tensile tester at room temperature (Figure 4B). SH:IA (1:1) showed the highest modulus (33 MPa) among all thiol-ene networks, correlated to its highest cross-linking density. A similar trend was also observed for elongation at break from 23 to 52% as the cross-link density decreases (Figure 4B). All thermomechanical data are summarized in Table 2.

**Figure 4:**
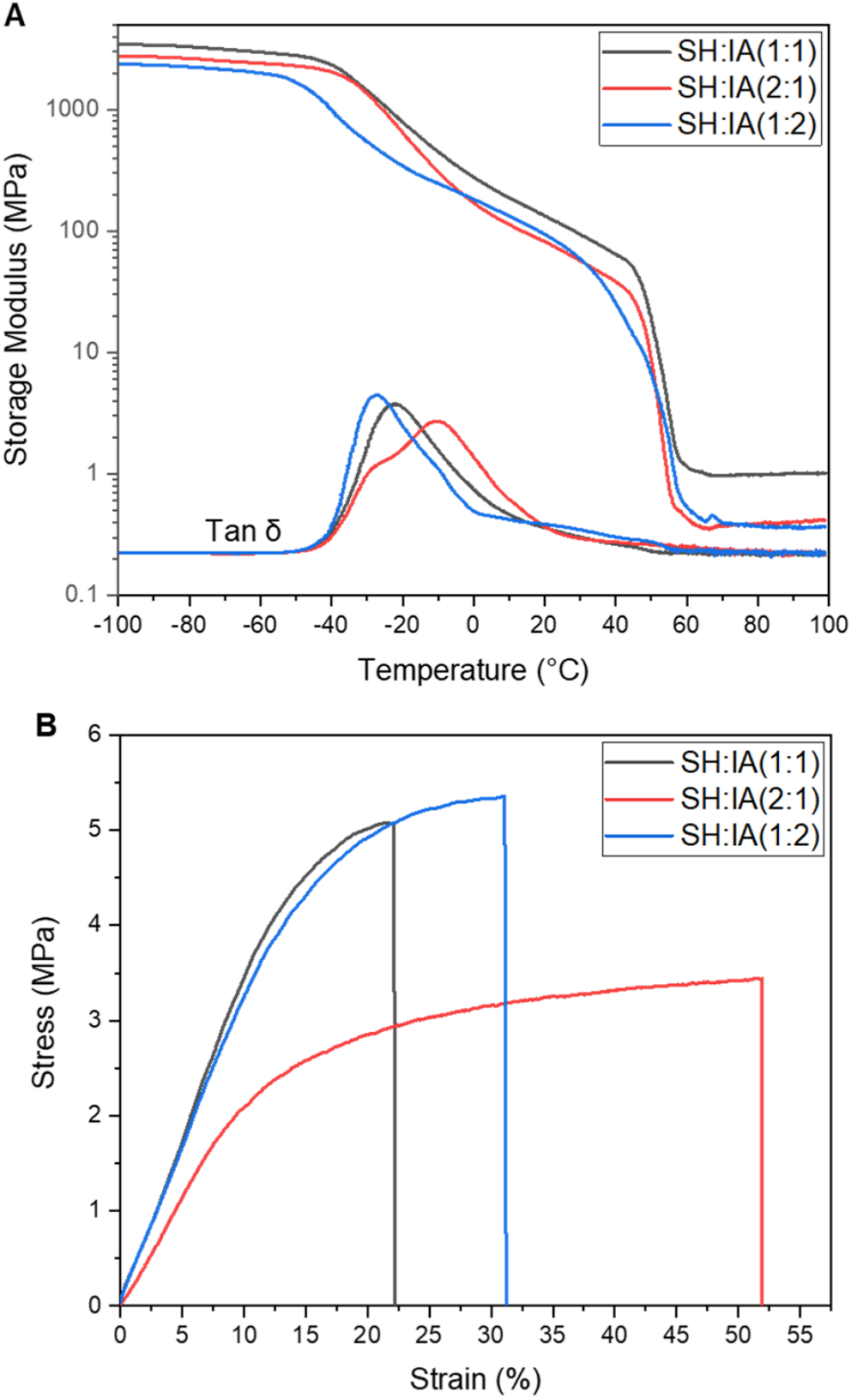
(A) Temperature-dependent storage modulus using DMTA in the tensile mode. Representative curves from *n* = 3. (B) Stress–strain curves measured in a static tensile test at room temperature. Representative curves from *n* = 5.

**Table 2:** Mechanical Properties of the Dry Cross-linked Networks (Dioxane)^a^

resin	*E*_glassy_ [MPa]	*E*_rubbery_ [MPa]	*T*_g_ [°C]	*E*_static_ [MPa]	elongation at break [%]	stress at break [MPa]
SH:IA (1:1)^b^	3311 ± 113	0.91 ± 0.19	–23.8 ± 1.6	33.1 ± 3.8	23.5 ± 1.9	4.8 ± 0.3
SH:IA (2:1)^b^	2719 ± 179	0.50 ± 0.11	–11.5 ± 2.0	17.6 ± 2.5	52.1 ± 14.4	3.4 ± 0.3
SH:IA (1:2)^b^	2287 ± 397	0.51 ± 0.24	–28.8 ± 2.5	25.6 ± 3.5	31.6 ± 10.8	5.2 ± 0.5
SH:IA (1:1)^c^				15.5 ± 2.7	52.6 ± 11.0	2.4 ± 0.3

a Young’s Modulus = *E*. *E*_glassy_, *E*_rubbery_, and *T*_g_ Determined from the DMTA. *E*_static_, Elongation at Break, Stress at Break Determined from Universal Tensile Tester.

b Solvent dioxane.

c Solvent GVL.

When the mechanical properties of these thiol-ene networks, particularly the SH:IA (1:1) formulation, are compared with similar systems synthesized from acrylated PCL of comparable molecular weight via free-radical polymerization, several notable trends emerge. The SH:IA = 1:1 thiol-ene network exhibits a higher tensile modulus, indicating greater stiffness, whereas the free-radical polymerized networks show enhanced elongation at break.^56^ This divergence is likely due to the fundamental differences in polymerization mechanisms: the step-growth nature of thiol-ene chemistry promotes more uniform and densely cross-linked network structures, thereby increasing stiffness but reducing extensibility.^37^ In contrast, chain-growth free-radical polymerization tends to produce more heterogeneous networks with longer, more flexible segments, resulting in greater stretchability.

### DLP 3D and 4D Printing of PCL–IA Polymers

Building on the photoreactivity of PCL–IA polymers, we explored their printability in DLP 3D printing for SH:IA (1:1). The resin formulations were used in dioxane or GVL as nonreactive diluents. Both resin formulations exhibited low viscosity (Figure S8), which is crucial for successful VAT printing. To control light penetration and diffusion, Sudan I was added to all formulations as a photoabsorber. Initial resolution trials were performed on the resin SH:IA (1:1) in dioxane and demonstrated satisfactory performance, with the system accurately reproducing fine features down to ∼0.5 mm (Figure S11). Both SH:IA (1:1) in dioxane and in GVL formulations were then printed into complex 3D geometries, with dimensions of *L* 8.2 mm × *W* 6.6 mm × *H* 5.8 mm and pore sizes up to approximately 0.6 mm. After removing the diluent, the printed geometry shrank to *L* 4.5 mm × *W* 4 mm × *H* 3.5 mm, while maintaining the same resolution (Figure 5). While a slightly lower print resolution was observed in GVL at close inspection, this would not be relevant in most applications. These results demonstrate the potential of the PCL–IA polymers as a sustainable option for VAT 3D printing.

**Figure 5:**
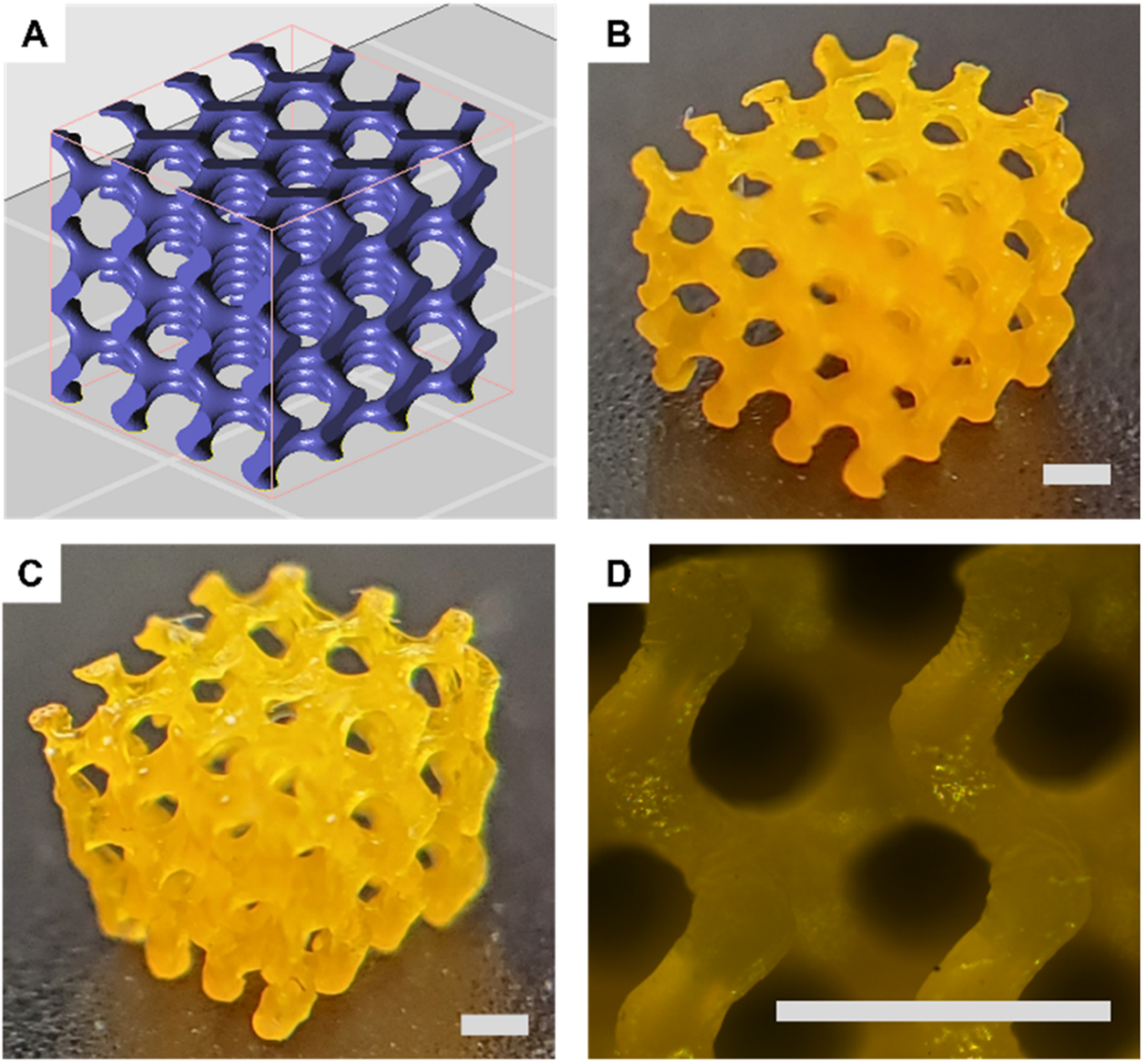
3D DLP-printed structures after removal of the diluent. (A) CAD design of the printed structures. Photos from DLP-printed structures based on (B) SH:IA (1:1) in dioxane (PCL–IA: 26 wt %, thiol cross-linker: 3 wt %, BAPO: 1.5 wt %, Sudan I: 0.05 wt %, layer exposure time 22 s) and (C) SH:IA (1:1) in γ-valerolactone (PCL–IA: 26 wt %, thiol cross-linker: 3 wt %, BAPO: 2 wt %, Sudan I: 0.06 wt %, layer exposure time 33 s); (D) zoom ×5 of SH:IA (1:1) in dioxane structure. Scale bar: 1.8 mm.

To further explore the potential of the PCL–IA base resin, a pH-responsive 4D network was printed by employing a hydrophilic 4-arm thiol-functionalized PEG (2300 g mol^–1^) (PEG-SH) cross-linker.^48^ This new resin formulation was printed into a gear shape (diameter of 10.5 mm) with a thiol-ene molar ratio of 1:1, using dioxane as a diluent. The printed object was then immersed in buffer solutions at three different pH levels (pH = 10, 7, and 4) for 24 h until equilibrium was reached. Notably, no structural disintegration was observed within this period in any of the tested pH buffers. The diameter of the printed PCL–IA increased from 10.5 to 13.5 mm when swollen in a pH 10 solution and then gradually decreased to 11.5 and 10 mm as the pH dropped to 7 and 4, respectively (Figure 6B). These results were corroborated by water uptake at the investigated pH values. At pH 10, the hydrogel exhibited the maximum degree of swelling, reaching 423 ± 4 wt %, which decreased to 280 ± 3 wt % at pH 7 and 108 ± 4 wt % at pH 4. This behavior is the consequence of the IA units in the printed network, which introduce cross-link points through their double bonds as well as free carboxylic acids responsive to protonation/deprotonation. Deprotonation of the IA cross-links at pH 10 results in a more hydrophilic and extended network, while at pH 4, the network collapses. At the higher network density at pH 4, PCL chains presumably can interact and form a semicrystalline structure, as suggested by their transition from transparent to opaque. When immersed in dioxane, the material exhibited repeatable pH responsiveness, demonstrating consistent behavior in a second cycle of swelling tests (Figure 6B).

**Figure 6:**
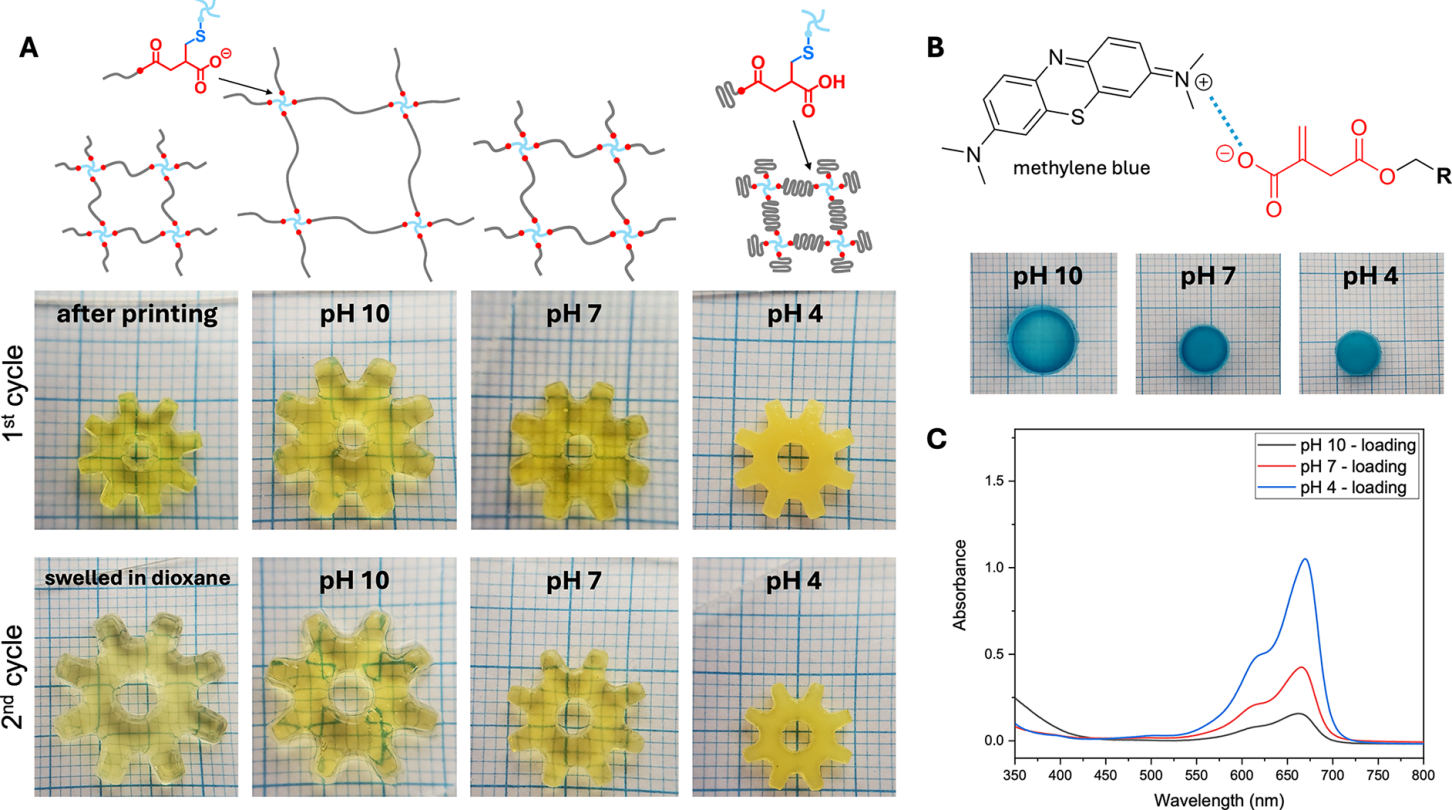
(A) Schematic of the network swelling as a function of pH and photos of the printed structure swelled in different buffer solutions for two consecutive cycles (swelling time 24 h). (B) Methylene blue uptake of cross-linked networks at different pH. (C) UV–vis spectra of methylene blue solution measured after immersion of cross-linked structures for 24 h, representing the nonabsorbed dye. Resin formulation for all samples: PCL–IA 19 wt %, PEG-SH 10 wt %, 1.5 wt % BAPO, and 0.03 wt % Sudan I (only for the 3D printed gears) in 1,4-dioxane.

While these experiments demonstrate that the incorporation of itaconic acid (IA) imparts 4D properties to the printed material, the free carboxylic acid groups also facilitate small molecule uptake. To investigate this, the absorption of methylene blue (MB), a cationic dye with a maximum absorption at 664 nm, was evaluated. Cross-linked hydrogels composed of PCL–IA and PEG-SH (1:1) were immersed in buffer solutions at pH 4, 7, and 10, each containing 7.5 μg·mL^–1^ MB. After 24 h of swelling at room temperature, the residual MB in solution was quantified via UV–vis spectroscopy (Figure 6C), allowing calculation of dye uptake. MB absorption was found to be pH-dependent, reflecting the ionization state of IA’s carboxylic groups. At low pH, limited absorption (1.8 ± 0.2 μg·mL^–1^) was observed, likely driven by hydrogen bonding between protonated carboxyl groups and MB amines, and compounded by network contraction.^57^ At high pH, deprotonation yields negatively charged carboxylates, promoting electrostatic interactions with MB, resulting in a significantly higher uptake (6.4 ± 0.1 μg·mL^–1^), further aided by increased network swelling (Figure 6B). These findings are in agreement with previous reports on IA-based hydrogels, which show enhanced affinity for cationic molecules at elevated pH due to carboxylate ionization.^58^

### Environmental Degradation

To assess the end-of-life impact of PCL–IA polymers, degradation studies were conducted on cross-linked lead sample SH:IA (1:1). Following standard protocols, accelerated degradation was carried out in an aqueous sodium hydroxide solution (0.2 M) at room temperature.^40,59^ Under these conditions, rapid hydrolysis of the ester bonds occurs. At each time point, the sample was removed from the solution, dried, and weighed. The results revealed that the sample degraded at a near-linear rate over approximately 20 h (Figure 7A). This rapid degradation is primarily attributed to the itaconic acid functionality, which, under basic pH conditions, can absorb more water. Notably, the gradual formation of microparticles during degradation suggests bulk disintegration (Figure S10). While accelerated degradation provides a quick laboratory assessment, it does not simulate environmental degradation. Therefore, the SH:IA (1:1) cross-linked network was also tested for microbial biodegradability following the OECD 301F test guidelines.^49^ The 301F method uses manometric respirometry, where a drop in pressure inside a closed measurement bottle is related to the quantity of oxygen taken up by the microbial community during oxidation/mineralization of the test substance and is used to calculate the percentage biodegradation over a period of time (Figure S12). The experiment was performed under buffered conditions (pH = 7.4) using a sewage-derived inoculum sourced from a local wastewater treatment works that primarily treats domestic sewage (Bracknell, UK). The samples were tested in duplicate and were compared with poly­(3-hydroxybutyrate) (PHB), which acts as a positive control and is known to be ‘readily biodegradable’ according to the OECD guidelines.^49^ PHB is a bioderived polyester that has been shown to be highly biodegradable by microbial populations present across a range of environmental compartments, including soil, compost, seawater, and sewage sludge.^60,61^ In our biodegradation test (Figure 7B), both PHB duplicates reached a percentage biodegradation of >60% after 6 days, in agreement with its classification as ‘readily biodegradable’, thus confirming that the inoculum has a sufficient abundance of microbes that are active in the breakdown of poly­(ester)-based materials. SH:IA (1:1) was also broken down by the microbial inoculum, with both duplicates achieving >60% biodegradation after 28 days. The biodegradation of polymers is influenced by various biotic and abiotic factors, as well as specific polymer characteristics, such as chemical composition, cross-linking density, molecular weight, and glass transition temperature (*T*_g_).^62^ Both the SH:IA (1:1) and PHB control exhibited a similar lag time of ∼ 2 days prior to a sharp inflection point where the curves start rising, inferring that a similar breakdown mechanism is occurring in both materials. The high prevalence of ester groups in the structures of both polymers means that this is likely to arise from esterase enzymes breaking up the polymer chains/network via ester hydrolysis, which then generates the smaller molecules that are ultimately mineralized to generate CO_2_. Importantly, ≥60% biodegradation within 28 days is the threshold required to classify a material as “readily biodegradable”, and this result demonstrates that the SH:IA (1:1) material can be broken down by microbes under more physiologically relevant conditions, suggesting minimal environmental persistence and impact at the end of its life cycle.

**Figure 7:**
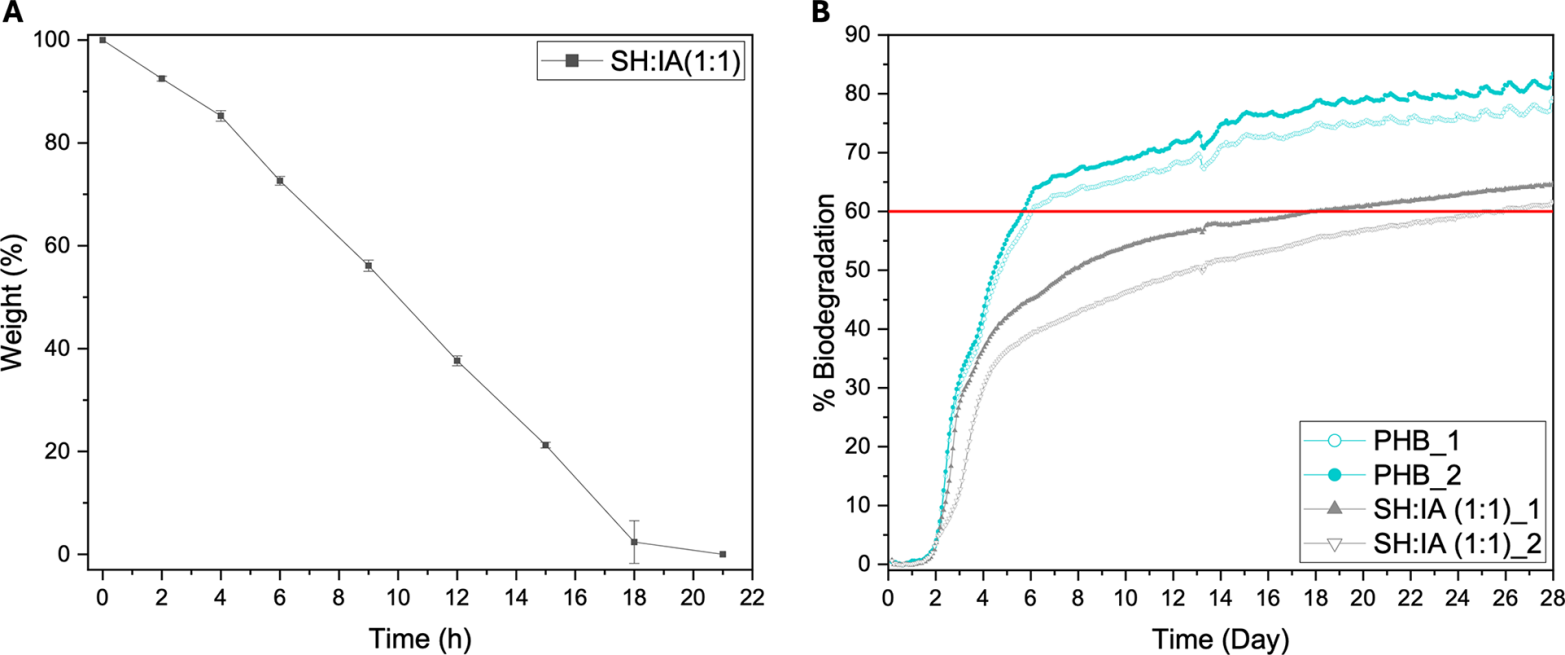
(A) Accelerated degradation in 0.2 M NaOH at room temperature. Error bars represent standard deviation (*n* = 3). (B) Biodegradation of the SH:IA (1:1) (red) cross-linked polymer network by microorganisms sourced from wastewater secondary effluent at pH 7.4. The data for the positive control compound Poly­(3-hydroxybutyrate) (PHB, blue) are shown for comparison.

## Conclusions

In this work, we demonstrated a pathway for producing functional and mechanically robust materials suitable for 3D and 4D printing. The study was guided by the use of sustainable materials and processes, addressing the environmental challenges associated with additive manufacturing. Within this context, we successfully developed a solvent-free synthesis route for itaconic-acid-functionalized polycaprolactone (PCL) with high end-group fidelity. The resulting PCL–IA showed excellent photoreactivity and printability, enabling the fabrication of complex 3D structures using dioxane and bioderived γ-valerolactone as solvents. This work highlights the multifunctionality of itaconic acid end-groups, which act not only as photocurable moieties but also as functional handles that impart responsive properties to the polymer network, as demonstrated by pH-responsive 4D structures. End-of-life evaluation revealed the potential for hydrolysis of cross-linked materials and microbial degradation in sewage water, underscoring the feasibility of biomass regeneration in a circular materials life cycle. Overall, the developed technology offers a straightforward and scalable route to functional 3D and 4D printable resins with properties suitable for applications in the biomedical space.

## Supplementary Material



## Data Availability

The data supporting this article have been included as part of the Supporting Information.

## Notes

NMR spectra of PCL and PCL–IA; photorheology, FTIR, and DSC of cured formulations; images of accelerated degradation; additional printed structures; UV–vis calibration curve of methylene blue. (PDF)
