# Supplementary material for: Polycaprolactone–Itaconic Acid Resins for Additive Manufacturing of Environmentally Degradable 3D and 4D Materials by Thiol-ene Photopolymerization
Source: Macromolecules. 2025 Aug 5;58(16):8887–97. doi: 10.1021/acs.macromol.5c01310 (PMC12392721; doi:10.1021/acs.macromol.5c01310)
Supplement: Supplementary file 1 [file ma5c01310_si_001.pdf]

Supporting Information

Polycaprolactone-itaconic acid resins for additive manufacturing of environmentally degradable 3D and 4D materials by thiol-ene photopolymerization

*Bo Li,[1,2] Gianluca Bartolini Torres,[1,3] Baptiste Martin,[4] Nicholas Taylor,[5] Eugen Barbu,[5] Annette Christie,[5] Andreas Heise[1,2,3]**

[1]Department of Chemistry, RCSI University of Medicine and Health Sciences, Dublin, D02 YN77, Ireland. [2]AMBER, The SFI Advanced Materials and Bioengineering Research Centre, RCSI, Dublin, D02 YN77, Ireland. [3]Science Foundation Ireland (SFI) Centre for Research in Medical Devices (CURAM), RCSI, Dublin, D02 YN77, Ireland. [4]Laboratoire de Chimie de Coordination, CNRS & Université de Toulouse (UPS, INP), 31077 Toulouse, France. [5]Syngenta, Jealott's Hill International Research Centre, Bracknell, UK, RG42 6EY.

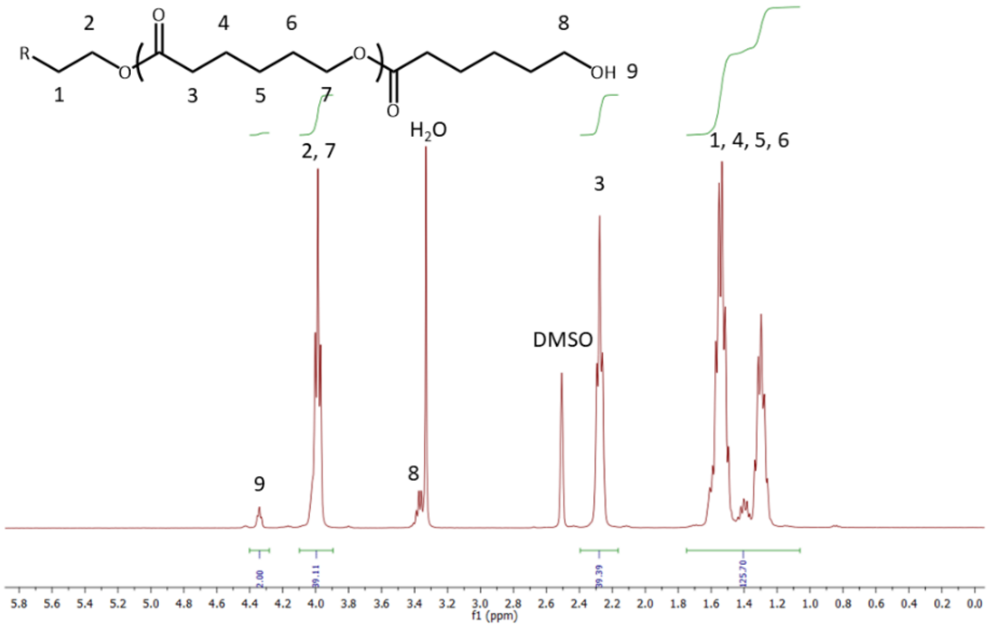

Figure S1. ^{1}H NMR spectra of PCL 2100 g·mol^{-1} (400 MHz, DMSO-d6).

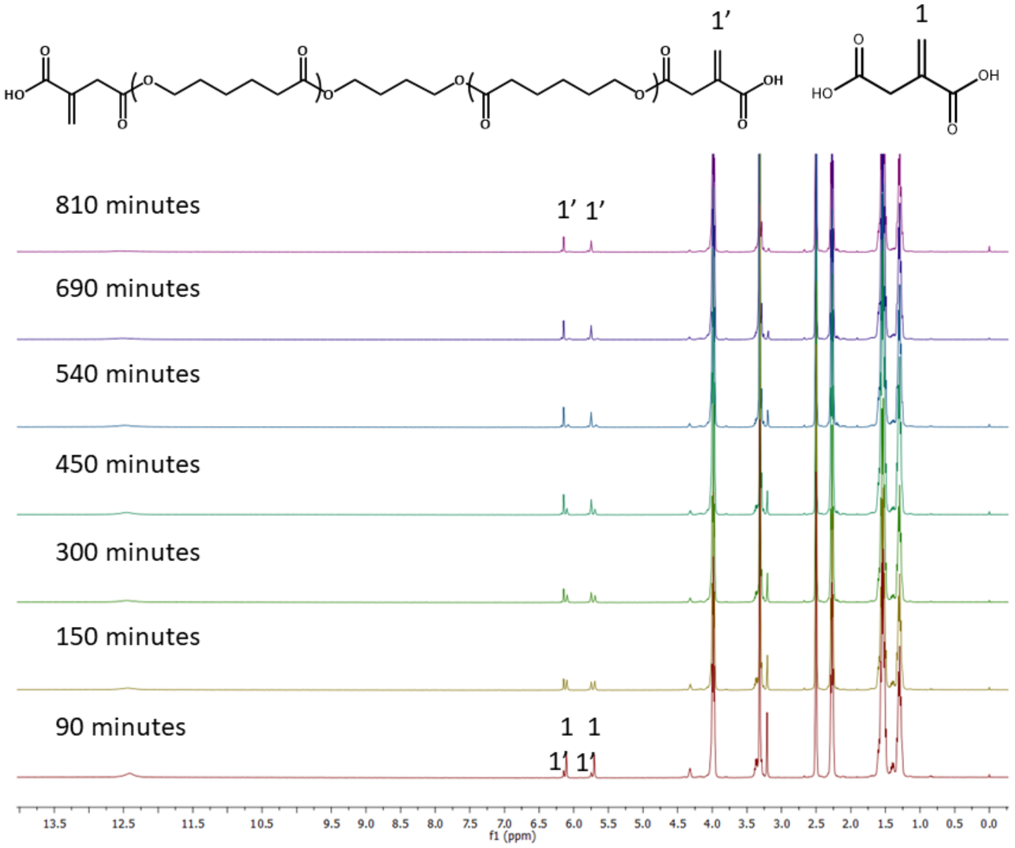

Figure S2. ^{1}H NMR spectra of samples retrieved after different time intervals during the esterification of PCL and itaconic acid at 140˚C (400 MHz, DMSO-d6).

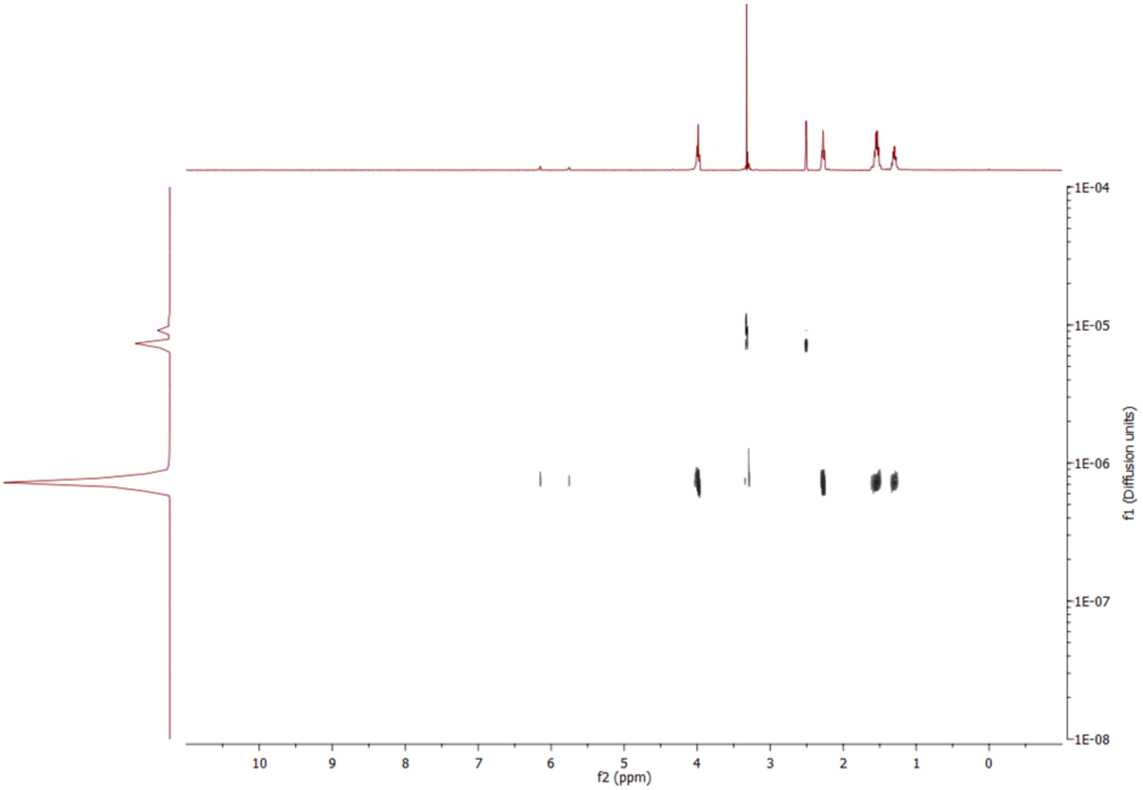

Figure S3. DOSY NMR of PCL-IA (400MHz, DMSO-d6).

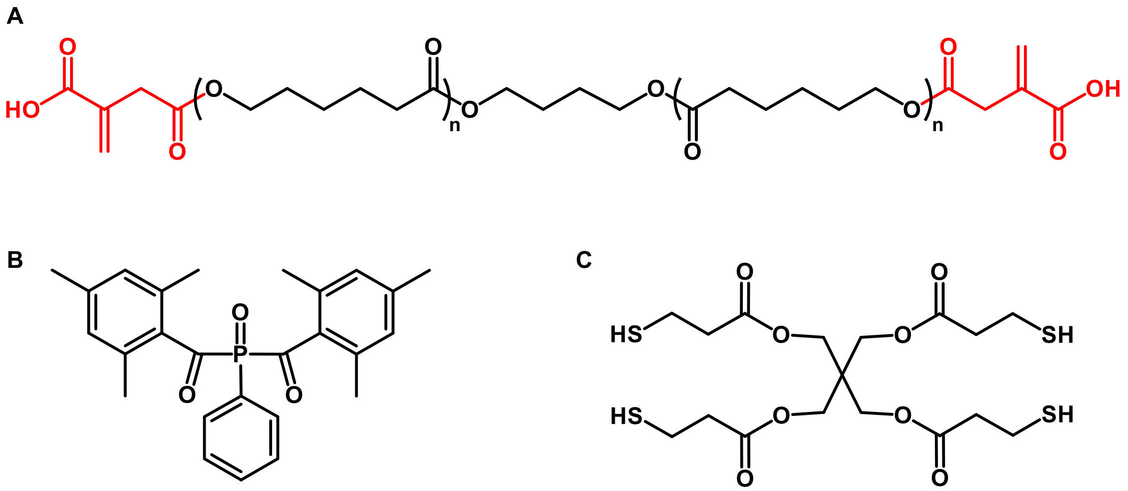

Figure S4. Resin formulation main components: a) PCL-IA, b) Phenylbis(2,4,6-trimethylbenzoyl)phosphine oxide (BAPO), c) Pentaerythritol tetrakis(3-mercaptopropionate) (tetra-thiol crosslinker).

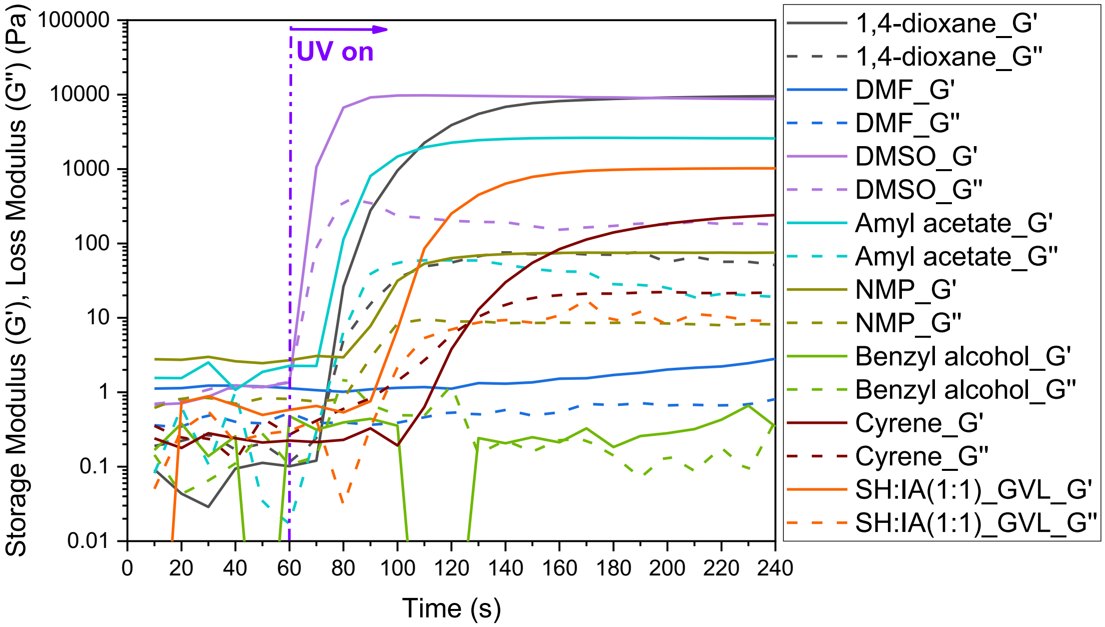

Figure S5. Photo-rheology graphs illustrating the formation of PCL-IA thiol-ene crosslinked networks in various solvents using the same formulation (PCL-IA + tetra-thiol at ~30 wt%, BAPO 1.5 wt%). For SH:IA(1:1) in γ-valerolactone (SH:IA(1:1)_GVL) BAPO 2%wt. UV light (405 nm) was turned on at 60 seconds (9 mW/cm^2).

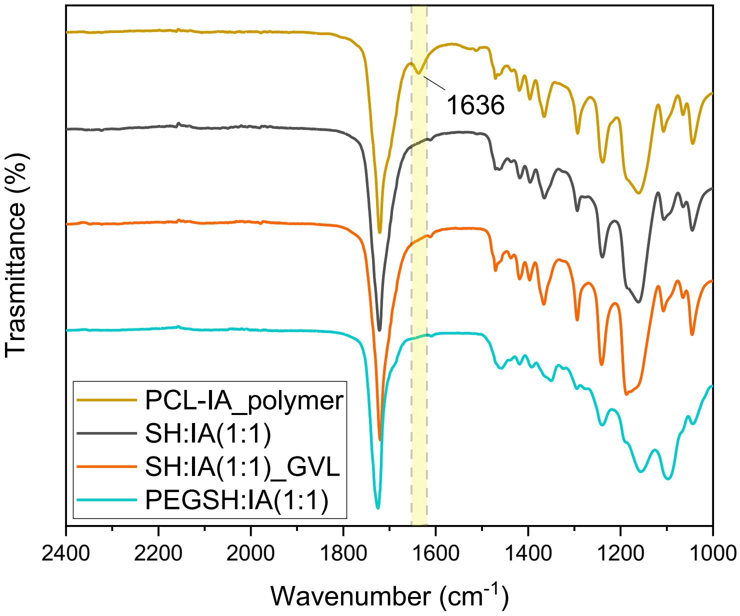

Figure S6. FTIR spectra of PCL-IA polymer, SH:IA(1:1) network, SH:IA(1:1)_GVL network and PEGSH:IA(1:1) network. The spectra were recorded after the removal of the diluent. The yellow region represents the itaconic acid double bond peak at 1636 cm^{-1}.

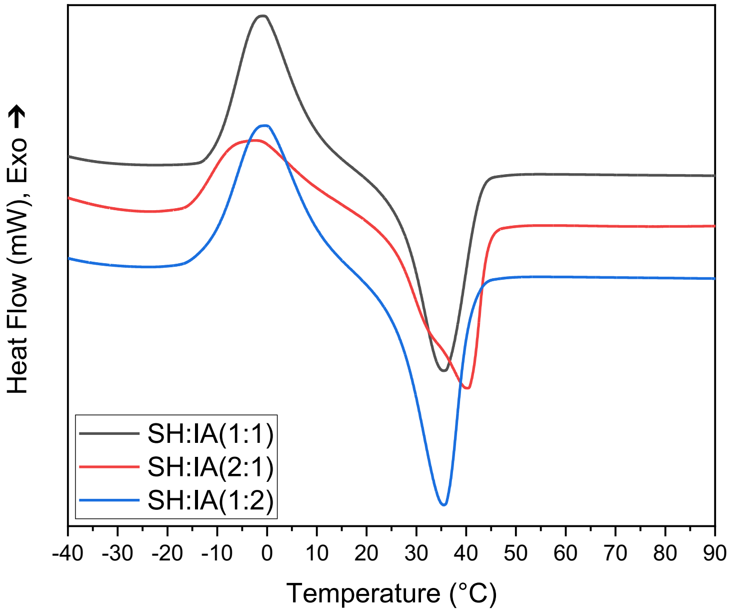

Figure S7. Differential Scanning Calorimetry (DSC) thermogram obtained from dry crosslinked SH:IA(1:1), SH:IA(2:1), SH:IA(1:2) networks. Heating ramp of the second cycle is showed. The exothermic peaks represent the cold crystallization. The endothermic peaks represent the melting.

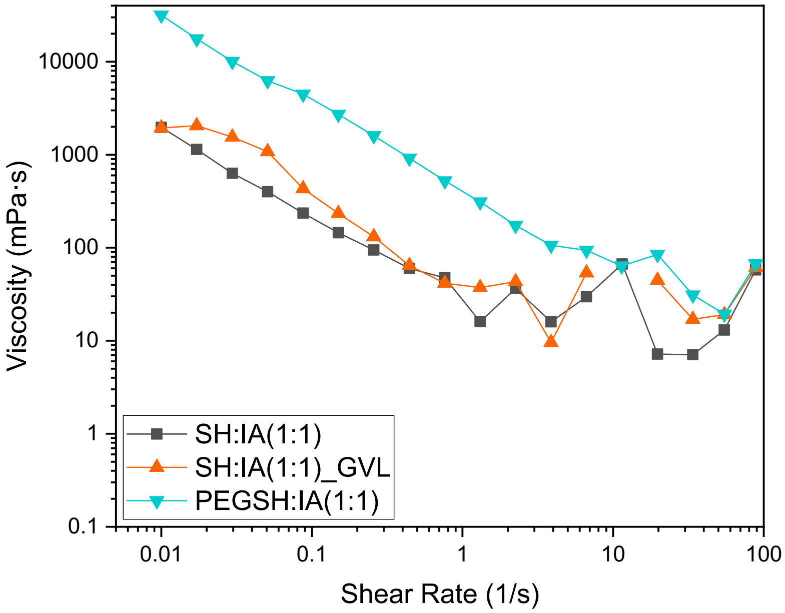

Figure S8. 3D printed resins viscosities for SH:IA(1:1) and PEGSH:IA(1:1) in 1,4-dioxane. SH:IA(1:1)_GVL in γ-valerolactone. Polymer plus crosslinker concentration at ~30 %wt.

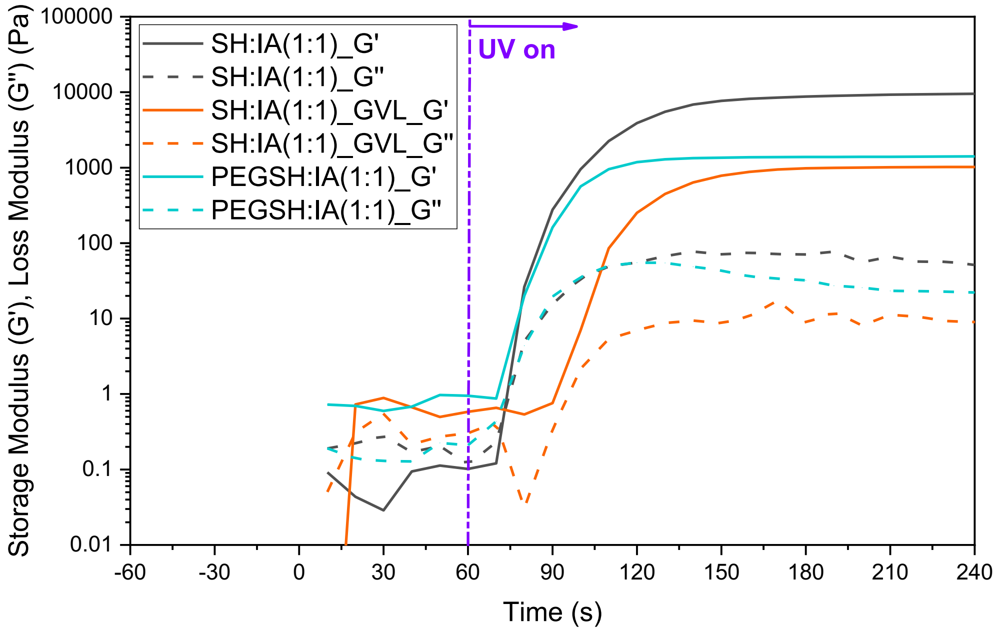

Figure S9. Photo-rheology graphs for resins printed by 3D DLP. UV light (405 nm) was turned on at 60 seconds (9 mW cm^{-2}). SH:IA(1:1) and PEGSH:IA(1:1) in 1,4-dioxane. SH:IA (1:1)_GVL in γ-valerolactone.

	0 hours	4 hours	9 hours	16 hours	30 Days
**SH:IA(1:1)** 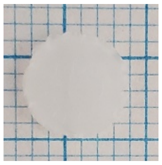	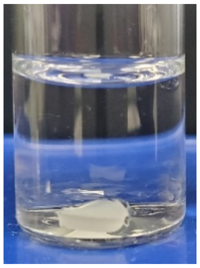	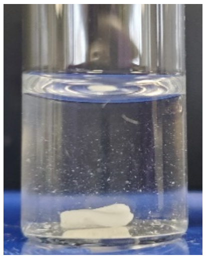	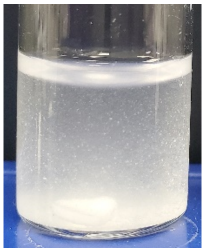	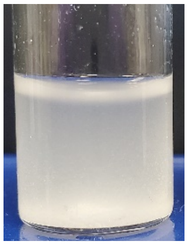	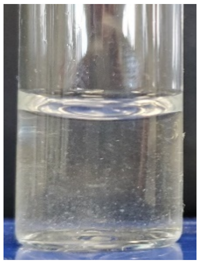

Figure S10. Degradation photos of SH:IA(1:1) in NaOH 0.2 M at room temperature at different time points.

Table S1. PEGSH:IA(1:1) network characterization[a]:

Polymer content	Diluent	Swelling test[b]	
		Gel fraction	Swelling degree
29 %	1,4-dioxane	73 .6 ± 1.3 %	10.4 ± 0.2

a) Photo-rheology Figure S9, FTIR Figure S6, viscosity Figure S8.
b) Swelling test was performed in 1,4-dioxane in triplicate.

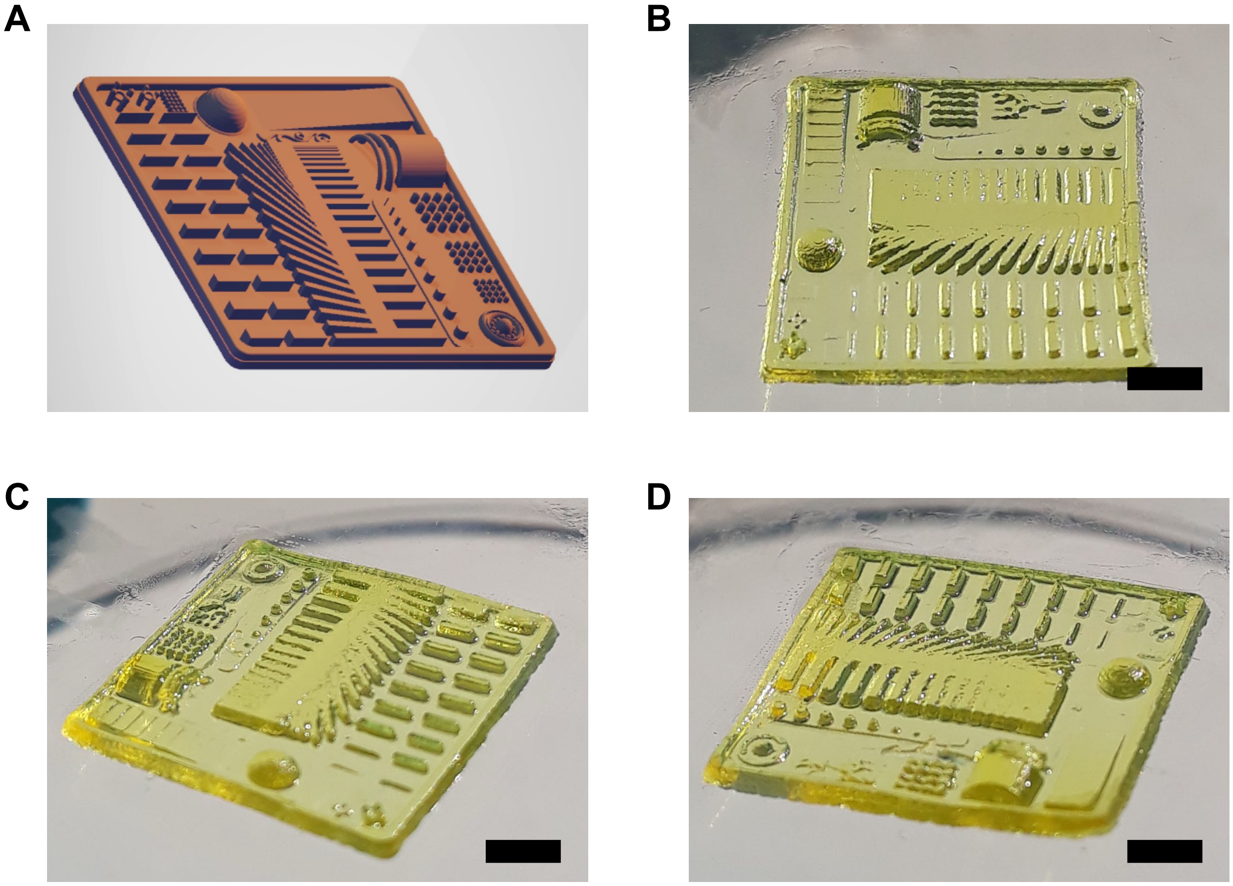

Figure S11: 3D DLP printed structures in 1,4-dioxane. A) CAD design of the printed structures. B, C, D) Photos from DLP-printed structures based on SH:IA (1:1) in dioxane (PCL-IA: 26 wt%, thiol-crosslinker: 3 wt%, BAPO: 1.5 wt%, Sudan I: 0.05 wt%, layer exposure time 22 s and. Scale bar 2.9 mm.

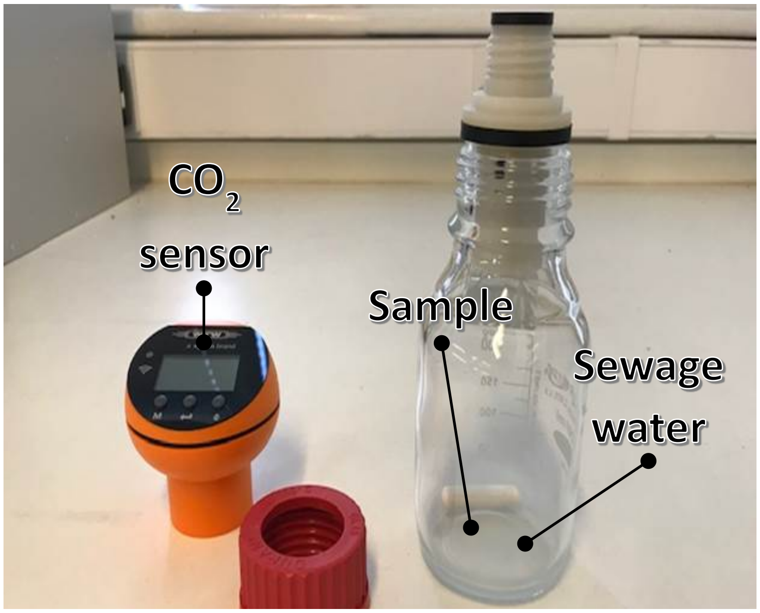

Figure S12. Manometric respirometry test measuring system OxiTop (WTW, Germany) used to determine the biodegradability.

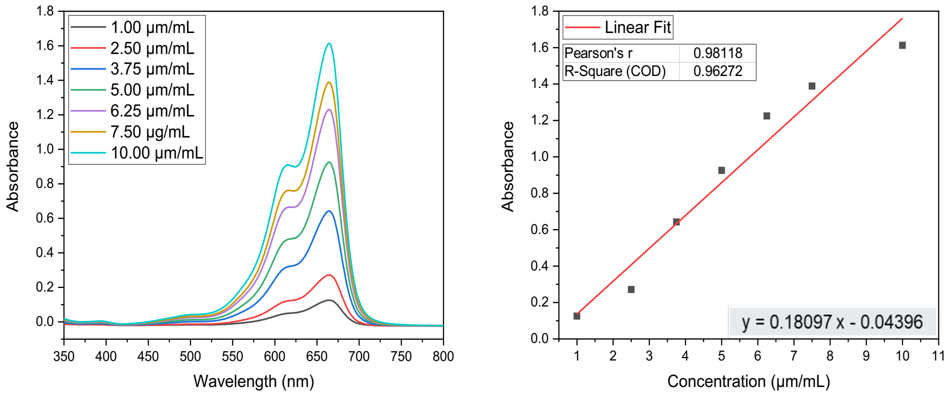

Figure S13: UV-Vis spectra of methylene blue at different concentrations in water and the calculated calibration curve.
